# A Literature Review of the Educational Gaps and Needs in Migraine Management: An Asian Perspective Within a Global Context

**DOI:** 10.7759/cureus.75337

**Published:** 2024-12-08

**Authors:** Siew Mooi Ching, Fung Lin Yong, Hsiao Wei Jao, Jecyll Santiago-Dayanghirang, Salil Prakash Shinde, Sajita Setia

**Affiliations:** 1 Family Medicine, Universiti Putra Malaysia, Serdang, MYS; 2 Medical Affairs, Pfizer Malaysia, Kuala Lumpur, MYS; 3 Medical Affairs, Pfizer Taiwan, Taipei City, TWN; 4 Medical Affairs, Pfizer Philippines, Manila, PHL; 5 Medical Affairs, Pfizer Hong Kong, Hong Kong, HKG; 6 Health and Medical Education, Transform Medical Communications Limited, Auckland, NZL

**Keywords:** asia, clinical practice guidelines, collaborative care, continuing medical education, educational needs, gaps in care, headache disorders, integrated care, medication overuse headache, migraine

## Abstract

Migraine is a common neurological disorder that presents considerable challenges to healthcare systems worldwide. These changes are especially relevant in rapidly developing regions such as Asia, with an increasingly productive population and ongoing advancements in healthcare systems and infrastructure. Despite its substantial impact, migraine management remains inadequate, potentially due to deficiencies in medical education. We hypothesized that significant gaps in basic and advanced medical training and continuing professional education contribute to the suboptimal management of migraine in various healthcare settings across Asia. A comprehensive literature review was conducted using PubMed and Google databases. The search focused on cross-sectional studies published in English from inception until September 2024 that examined educational needs among medical trainees and clinicians and clinical gaps in migraine management in Asia. These studies were then contextualized within a global perspective. The review identified significant shortcomings in migraine education at all levels of educational training in Asia, which also translated to poor management of migraines in clinical practice. Undergraduate medical curricula in Asia inadequately address headache disorders, while postgraduate training programs provide insufficient guidance in headache management, especially for complex cases. Primary care clinicians exhibited variable levels of understanding and frequently incorrectly diagnosed and managed migraine. Additionally, many Asian countries lack standardized clinical practice guidelines (CPGs) and specialized training programs for headache management. A multidimensional approach is required to tackle the pre-existing educational and clinical practice limitations. The approach should include improving the medical school curriculum, providing focused continuing medical education programs or developing migraine modules for primary care physicians (PCPs), and developing region-specific CPGs. Besides educational initiatives, integrating and coordinating systems of care, where primary and specialist services complement each other, are crucial for improving patient care. Robust education combined with comprehensive referral and linkage protocols ensures continuity of care across healthcare levels. Moreover, collaboration, communication, and cooperation among healthcare providers (HCPs) and organizations are vital to enhancing the quality of life and productivity of migraine patients in the region. A synergistic approach that combines robust collaboration with innovative educational delivery can catalyze the widespread adoption of evidence-based medicine (EBM).

## Introduction and background

As per the Global Burden of Disease (GBD) Study 2019 report, headache disorders, with migraine as the primary contributor, were the 14^th^ leading cause of disability-adjusted life years (DALYs) globally across all ages and genders [1]. These disorders accounted for 46.6 million years lived with disability (YLDs), representing 5.4% of total YLDs, of which 88.2% were due to migraine [1]. This is comparable to 46.6 million people each losing a year of healthy life [2]. Among the top four causes of YLDs, migraine ranked second, following low back pain. Among females aged 15-49 years, migraine was the topmost contributor to YLD, indicating its alarming burden on young women [2]. Similarly, in the GBD 2021 report, headache disorders were among the top three leading causes of YLD and accounted for 48 million YLDs, which disproportionately affected young women [3]. When comparing YLD figures, headache disorders ranked among the top three non-communicable diseases contributing to YLD, following low back pain (70.2 million YLDs) and depressive disorders (56.3 million YLDs) [3]. This recent report also highlighted no sign of improvement in the burden of these disorders between 2010 and 2021, with a 0.3% increase in age-standardized YLD rates predominantly affecting females aged 15-49 years [3]. Given the statistics, the authors of the GBD publication have urged decision-makers to pay closer attention to the burden of migraine, especially among young women [1]. Yet, despite the globally recognized high burden of migraine, healthcare provision for this condition remains suboptimal worldwide. A significant number of individuals suffering from headache disorders do not receive medical evaluations, as many do not seek formal assessments from healthcare providers [4].

A significant rise in migraine incidence, prevalence, and DALYs was observed globally among youths and young adults aged 15-39 years. From 1990 to 2021, the global prevalence of migraine in this age group surged by 39.5%, from 425.6 million cases to 593.8 million cases [5]. However, regionally, certain areas bear the greater brunt of this global escalating burden and rising prevalence [5]. The global estimated annual percentage change (EAPC) over the past three decades was 0.09, with Eastern Europe, Central Europe, and Western Europe recording an EAPC of 0.08, 0.06, and 0.03, respectively. East Asia experienced the most rapid increase in prevalence and DALY for migraine in the young population, with an EAPC of 0.31, while Southeast Asia (SEA) witnessed the steepest decline in prevalence (EAPC: -0.04) [5]. However, high-income countries in SEA, like Singapore, also saw an upward trend in prevalence, likely due to better healthcare systems, improved diagnosis, and the stressful, high-paced lifestyles typical of these nations [5]. Ge R et al. published the epidemiology of migraine from non-high-income East Asia and SEA based on the GBD 2019 report in 2023 [6]. There were approximately 195.7 million patients with migraine in East Asia and 113.4 million in SEA, with Thailand recording the highest age-standardized prevalence and YLD rates for migraine at 19,349 and 645 cases, respectively, per 100,000 population [6].

Migraine management in Asia: what we know and don't know

Asia is currently experiencing rapid economic and social transformation, accompanied by significant shifts in its demographic structure with a relatively higher productive population and developments in the healthcare system and infrastructure [7]. Understanding gaps in migraine education with a global outlook in these varied contexts is crucial for developing regionally appropriate strategies for the management of migraine in Asia. The most recent global estimates suggest that migraine affects 14%-15% of the population [8]. As expected, these figures likely underestimate the true extent of the issue [8]. Surveys employing the International Headache Society (IHS) classification criteria reveal that migraine is a prevalent disease in Asia, with rates slightly lower but comparable to those reported in Western countries [9, 10]. Nonetheless, the geographical or ethnic variations in migraine or other headache types might be less pronounced if identical epidemiological methodologies and case definitions were applied consistently in surveys across different global regions [9]. Moreover, various specific factors contribute to the high burden of headaches in Asia [11]. These include [11]: densely populated urban centres that increase stress, pollution, and lifestyle-related triggers; socioeconomic disparities that limit access to adequate healthcare resources and headache specialists; environmental factors such as air and noise pollution and climate variations that exacerbate headaches; genetic and ethnic diversity leading to variations in headache prevalence and presentation; and significant disparities in healthcare infrastructure, including a shortage of neurologists, limited headache services, and inadequate training programs for healthcare professionals.

Data on the burden, diagnosis, and management of migraine in Asia from January 1, 1988, to January 14, 2019, were systematically collected by Takeshima T et al. [10]. However, their search specifically targeted only studies from China, Japan, and South Korea. Although migraine was consistently associated with substantial disability and adversely affected the quality of life, data on the economic burden and clinical management of migraine were scarce, more so for children. Across these countries, there was a noticeable trend of low disease awareness and diagnosis rates. For instance, in China, 52.9% to 68.6% of individuals with migraine had previously consulted a physician, yet 37.2% to 52.7% diagnosed with headache had not been identified as migraine sufferers, and only 13.5% to 18% had a prior migraine diagnosis. In Japan, 59.4% to 71.8% of migraine sufferers had never sought medical advice, only 1.3% to 7.3% regularly consulted physicians for their headaches, and a mere 11.6% were aware of their migraine condition. The research also indicated a high usage of over-the-counter (OTC) medications and a low usage of prescription medications for migraine in these countries. The main reasons for patients not consulting a physician were that the respondents could tolerate their symptoms, the OTC medication was effective, the headache improved spontaneously, or the inability to miss work. The study highlighted unmet needs for better detection and treatment of migraine as it imposed a substantial humanistic burden [10]. For example, in China, assessments of health-related quality of life revealed that migraine negatively impacts almost all Short Form 36 (SF-36) domains, while the population-based studies in Japan and South Korea found that approximately one-third of individuals with migraine experienced moderate to severe levels of disability [10]. The results also translate to the resulting economic burden [12-15]. A population-based door-to-door survey from China revealed that individuals with migraine reported an average of three missed work or housework days, four impaired work days, and nine impaired housework days, resulting in an estimated US $39.4 billion in indirect costs from lost productivity. Combined, the total annual cost of migraine was estimated to be US $47.8 billion [10, 13]. A cross-sectional online survey conducted among full-time employees in Singapore found that episodic migraine resulted in a substantial economic burden, costing the country approximately Singapore $1 billion (US $0.75 billion) in 2018 [14]. Most of these costs (82.4%) were due to lost work productivity, highlighting the significant impact of migraine on both individuals and the economy [14]. A retrospective matched case-control study in Taiwan revealed that patients with refractory migraine incurred significantly higher average total medical costs over one year following their initial visit compared to non-migraineurs (US $1,783 vs. $825) and those with other migraine types (US $1,682 vs. $1,181) [15].

Despite the availability of effective treatments for headache disorders, including a wide range of acute and preventive options developed over the past 30 years, the global burden of migraines remains unacceptably high [16]. This persistent challenge is largely due to failures in health policy and inadequate care delivery systems, particularly at the primary care level, where general practitioners (GPs) often feel uncomfortable diagnosing and managing primary headache disorders. Integrating headache care into primary healthcare services and ensuring appropriate access to secondary and tertiary care when necessary are crucial steps [17]. Additionally, in Asia, particularly in low- and middle-income countries, there is a significant shortage of neurologists who are crucial in managing hard-to-treat headache disorders. The 2017 World Health Organization Atlas of Country Resources for Neurological Disorders reported a median of only 0.04 neurologists per 100,000 population in the SEA, hugely contrasting with the median of 6 neurologists per 100,000 population in the European Region [18]. The scarcity of neurologists also limits the capacity to train other GPs in headache management [18].

To overcome the challenges in diagnosing and effectively managing migraines, suggested potential strategies include identifying educational and management deficiencies, implementing educational programs for healthcare workers, introducing a tiered system for headache care, developing diagnostic and management algorithms tailored to local contexts, and adopting a stepped approach to headache treatment [18]. Migraine frequently goes unrecognized, is incorrectly diagnosed, and receives insufficient treatment in primary care environments [19]. For example, in an ideal scenario, chronic migraine (CM) should be diagnosed in primary care settings; however, less than a quarter of individuals with CM are correctly diagnosed [20]. A significant obstacle to correct diagnosis and delivering effective care is the lack of adequate formal and continuing education and training among healthcare professionals [17], and it is important to understand the gaps, particularly in Asia, within a global context.

We hypothesize that significant gaps in both basic and advanced medical training, along with insufficient continuing professional education, contribute to the suboptimal management of migraine across various healthcare settings in Asia. This narrative review aims to identify and analyze the educational needs among medical trainees and clinicians and evaluate the clinical practice deficiencies in migraine management in Asia, contextualized within a global perspective.

## Review

Methodology

We conducted a literature search to identify and analyze the educational needs and gaps in migraine management among healthcare providers (HCPs) using a semi-systematic search strategy, utilizing both PubMed and Google as our primary search engines. A semi-systematic review approach has been regarded as an optimum strategy for identifying knowledge gaps in the literature [21]. We combined Medical Subject Headings (MeSH) and free-text terms on PubMed to construct our query. The search terms included variations and combinations of keywords such as “migraine”, “headache disorders”, “education”, “knowledge”, “health care workers”, “primary care”, “Asia”, “gaps in care” and “needs assessment”. The search strings were formulated using Boolean operators (AND, OR) to combine the terms effectively on PubMed. The keywords for geographic focus, such as ‘Asia’ and specific country names within Asia, were also included with repeat searches. We adopted flexibility in modifying search terms based on initial findings and repeated the search process after retrieving relevant articles. After each search iteration, we analyzed the results to identify any additional relevant terms or subject headings that could enhance the search. If certain relevant articles were identified, we examined their keywords and subject headings to refine our search terms further. This iterative process was repeated multiple times to ensure that emerging concepts and terminologies were captured. The search included articles published from inception until September 2024. Reference lists of key articles were reviewed to identify additional relevant studies not captured in the database searches. Table 1 lists the eligibility criteria for data extraction.

**Table 1:** Eligibility criteria for data extraction The inclusion criteria focused on articles published in English, with a specific emphasis on studies conducted in or relevant to the Asian context. The search included articles published from inception until September 2024.

Inclusion criteria	Exclusion criteria
Population	
Studies involving medical trainees (undergraduate and postgraduate), and practising clinicians involved in migraine management.	Studies focusing solely on patient populations without addressing the educational aspects for healthcare providers.
Focus	
Studies examining educational needs, knowledge gaps, attitudes, or practices related to migraine diagnosis and management among healthcare providers.	Articles that do not address educational needs or gaps in migraine management, such as those focusing exclusively on clinical studies of migraine treatments without an educational component.
Study design	
Cross-sectional surveys and related observational studies that provide empirical data on educational gaps or deficiencies in migraine diagnosis or management.	Editorials, commentaries, letters to the editor, case reports, and reviews without original data.

Unmet educational needs on migraine education among medical students

In undergraduate medical education, a remarkable unmet need for prioritizing headache disorders within the curriculum was found in a study from Singapore [22]. Over half of the participants (55.1%) had not received formal instruction in taking a complete headache history, and a vast majority (90.6%) had never attended a headache sub-speciality clinic. On average, they had been exposed to headache disorders for 5.69 hours [22]. The students from this study reported antidepressants as the most frequent incorrect option for the abortive treatment of migraine. Another study published in 2005 revealed significant insights into headache education in medical training in the United States [23], which can be connected to the more recent findings from medical students in Singapore [22]. Medical schools considered their training adequate, but neurology and family practice residency program directors felt that residents were inadequately prepared in headache management upon entering their programs [23]. This suggests a disconnect between undergraduate training and the expectations of residency programs. Common topics like diabetes received more extensive coverage than headaches in medical schools, indicating a potential underestimation of the importance of headache disorders in medical education [23]. The under-prioritization of education for headache disorders in medical schools leads to ineffective management and poor patient outcomes, as primary care providers (PCPs) often lack the necessary expertise to treat these common conditions effectively. It is crucial to emphasize the importance of headache disorder training at this foundational level, especially considering that most headache cases are handled in primary care settings [24,25]. Furthermore, a significant gap exists in understanding and identifying different types of headaches. This lack of basic knowledge necessitates the urgent need to integrate comprehensive headache education into undergraduate medical programs, ensuring future healthcare professionals are well-equipped to address the current gaps in care [25].

Table 2 provides key insights and findings from cross-sectional studies highlighting educational shortcomings and needs in migraine management for medical students.

**Table 2:** Summary of identified cross-sectional studies on educational needs and gaps in migraine management among medical students ICHD: International Classification of Headache Disorders

Key insights collected	Key findings
Ong J et al., 2017 [22]: Knowledge base and perception of Singapore medical undergraduates in headache training and education	
Medical undergraduates demonstrated considerable uncertainty regarding local and international guidelines on headache management, with significant concern arising from their inability to name a single abortive treatment correctly. This confusion extended to differentiating between abortive and prophylactic treatments.	One hundred and twenty-seven final-year medical students completed the survey; 55.1% of the respondents did not receive formal teaching on taking headache history. 96.1% were unfamiliar with locally published clinical practice guidelines; 74.9% were unfamiliar with the 3^rd^ edition of ICHD; 47.2% were unfamiliar with medication-overuse headache as a disease entity. Antidepressants were most frequently reported as incorrect options for abortive migraine treatment; 18.9% were unable to name a single abortive treatment for migraine correctly; 62.2% self-reported their exposure to headache management and diagnosis as inadequate; one out of five rated opioids as an abortive choice.
Gallagher RM et al., 2005 [23]: Perceived adequacy of headache education among medical students, neurology, and family practice residents in the United States	
Undergraduate medical education in headaches was limited. While medical schools perceived their training on the subject as adequate, both family practice and neurology residency program directors believed that incoming residents were inadequately prepared for headache education upon entering their programs.	Survey response rates were 35%– 40% of the total sent to all allopathic and osteopathic medical schools, 200 family medicine residencies, and all 126 neurology residencies. Common topics like diabetes were more extensively covered than headaches. Medical school lecture hours on headache education ranged from 0 (4%) to >5 (24%), with 92% having no plans for an increase at that time.

Unmet educational needs on migraine education among residency students

A recent study in Saudi Arabia analyzed the challenges and barriers to headache training among neurology residents [26]. This study highlighted the critical issue of diagnosing headaches and the prevalent problem of medication overuse headaches due to the ease of obtaining OTC analgesics. It suggested ongoing headache education and comprehensive academic training as remedies. Notably, more than half of the residents self-rated their knowledge as good or very good for common headaches but less so for complex types like trigeminal post-traumatic headache or autonomic cephalalgia [26]. A study from France, published in 2022, revealed that the International Classification of Headache Disorders-3 (ICHD-3) classification was insufficiently used by neurology residents [27]. The study highlighted the essential need for specific training programs in headache medicine, pointing out the inconsistencies in MRI requests and a lack of adherence to national recommendations. The study also found a significant gap in the understanding of medication overuse and the use of headache diaries [27]. Another 2022 publication from Denmark found that neurology residents' knowledge of headache disorders fell short of national and international standards, with a noted deficiency in formal education being a significant barrier [28]. Despite two-thirds of residents having formalized training in headache disorders, gaps were identified in diagnosis, management, and referral practices, and headaches ranked low in interest among sub-specializations [28]. Lastly, a 2016 survey among neurology residency program directors and chief residents in the United States showed that while there was a slight increase in headache-related teaching, many still felt the educational experiences in headache management were inadequate [29]. The survey revealed that only 26% of programs had a mandatory headache clinic with varying lengths of exposure, and a significant portion of respondents believed that more than four weeks of clinical experience in headaches is necessary for adequate preparation of neurology residents [29].

Table 3 summarizes the overall insights and key findings. It highlights a moderate advancement in understanding from the medical student level, where fundamental knowledge gaps are more prominent.

**Table 3:** Summary of identified studies on cross-sectional studies educational needs and gaps in migraine management among speciality/ family medicine training residents ICHD: International Classification of Headache Disorders; MOH: medication overuse headaches

Key insights collected	Key findings
Tawakul AA et al., 2023 [26]: Challenges and barriers to headache training among all neurology residents in Saudi Arabia	
Inaccurate diagnosis is the most challenging barrier in headache management. Additionally, the ease of obtaining over-the-counter analgesics has led to high rates of MOH. Ongoing headache education and comprehensive academic training are recommended to enhance patient care.	Two hundred and twenty-seven neurology residents completed a questionnaire-based survey. More than half of residents self-rated their knowledge as good or very good for migraine (62.6%), tension-type headache (60.4%), and cluster headache (55.5%). Less than half of residents self-rated their knowledge as good or very good for trigeminal post-traumatic headache (39.6%), autonomic cephalgia (40.5%), and MOH; 44.3% of the residents collaborated well with general physicians (GPs) for headache patients. Only 8.3% of residents referred >30% of headache patients to specialized headache centres.
Baltramone M et al., 2022 [27]: Knowledge of headache medicine among neurology residents in France	
ICHD-3 classification is insufficiently utilized in clinical practice, along with deviations from national recommendations and unnecessary MRI requests. While medication overuse is often considered, its risks are less frequently explained to patients. Additionally, headache diaries are generally not used for diagnosis, though they may be adopted during follow-up.	A total of 54 residents completed the survey. Different types of training programs in France did not impact respondents' knowledge of the management of episodic migraine. Only 21.5% used the ICHD‐3 criteria to diagnose headaches. Only 29% had read the French guidelines for diagnosis and management of migraine, and only 83.3% knew that an aura could occur during the headache phase.
Do TP et al., 2022 [28]: Barriers and gaps in headache education for neurology residents in Denmark	
Knowledge of neurology residents in Denmark for headache disorders does not meet the expectations from the national and international recommendations. There is a relative lack of formal education, serving as a barrier to adequate headache care.	Fifty of 150 residents completed the survey. Two out of three of the respondents had prior formalized training in headache disorders. Gaps were identified in all explored domains, including diagnosis, management, and referral patterns along with inconsistent use of guidelines and diagnostic criteria. Headache was ranked second to last out of six sub-specializations in interest.
Ahmed ZA et al., 2016 [29]: Survey among program directors and chief residents on headache didactics and clinical training in neurology residency programs in the United States	
Despite a recent slight increase in headache-related teaching within neurology residency programs, many program directors and chief residents still believe their programs lack sufficient educational experience in headache management. There is still a need to enhance didactic and clinical exposure dedicated to managing headache disorders in approximately one-quarter of these programs.	One hundred and thirty-three neurology resident program directors and 213 chief residents were surveyed; 26% of neurology residency programs reported having a mandatory headache clinic. Within these programs, 35% offered < 2 weeks, 54% provided two to four weeks, and 12% offered > 4 weeks of clinical exposure to headache; 51% of program directors and 40% of chief residents believed that > 4 weeks of clinical experience in headache is necessary for adequately preparing neurology residents.

Perspectives from specialists and PCPs

A cross-sectional study was conducted by Roxas A Jr et al. by interviewing neurologists between August 2020 and February 2021 from the 21 member countries of the Asian Oceanian Association of Neurology and three other Asian nations. This study revealed significant gaps in migraine management and education in Asia [30]. Only 54% of these countries have a dedicated headache council, and 75% lack a subspecialty training program for headaches. Prevalence studies on migraines are available in 14 countries, but only 10 out of 24 have clinical practice guidelines. Most countries have access to non-specific migraine drugs, but non-oral triptans are available in only eight countries, and monoclonal antibodies for migraine prophylaxis in 50%. The survey highlighted that migraine is commonly underdiagnosed and under-treated, especially by non-neurologists. A clear need for more educational focus on primary headaches in medical schools was highlighted, with the current average lecture time being only 2.77 hours, significantly less than the reported average time for such formal education in low-income countries (four hours). In terms of healthcare delivery, there is a disparity between primary and secondary care, with an emphasis on the need for PCPs to have more professional knowledge and resources in differential diagnosis and management of headaches [30]. This study sought the educational needs of specialists but mainly pointed out the flaws in basic foundational medical education for headaches and migraines. Another study by Roxas A Jr et al. highlighted several deficiencies in practice patterns among neurologists in the Philippine Neurological Association 2018 register in the Philippines [31]. The findings revealed that only 14.7% used the Migraine Disability Assessment Scale to assess migraine disability. While 62.2% advised the use of headache diaries, a significant 77% requested neuroimaging for patients with over three years of recurrent severe migraine, suggesting a reliance on diagnostics that may not add value.

Additionally, 51% never or seldom prescribed triptans for recurrent episodic moderate to severe migraine on the first outpatient consult, and only one-third provided anti-nausea medications for moderate to severe attacks. Only about one-third prescribed migraine prophylaxis for at least 12 weeks or longer. The study emphasized the need to promote using ICHD criteria for diagnosing migraines to reduce unnecessary costs associated with electroencephalography (EEG), which also provides limited value in diagnosis. Furthermore, a pressing need to counsel patients on medication overuse was highlighted, as self-medication and overuse of nonsteroidal anti-inflammatory drugs (NSAIDs) are common due to the high cost of triptans. In the Philippines, significant gaps in migraine management arise from limited training opportunities and a shortage of headache specialists, necessitating neurologists to seek fellowships in headache medicine overseas. Only a minority of neurologists from the Philippines have had exposure to specialized headache clinics during their training, primarily due to the lack of such specialists in the country. Migraine management practice was also investigated almost two decades ago among neurologists in Taiwan [32]. This study found that while a significant majority (88.5%) considered headaches as an important part of their practice, most lacked sufficient knowledge about medication overuse headaches (MOHs), including the fact that common medications like ergotamine, acetaminophen, and triptans are frequent causes. A substantial proportion relied on the unnecessary use of diagnostic tools like neuroimaging (65.0%) and EEG (44.7%) for diagnosis. A study published in 2005 surveyed neurology residency program directors and chief residents in the United States [33]. This study also highlighted the importance of migraine education in medical schools. Out of 119 respondents, including chairs and training directors from 95 institutions, only 29% felt that the topic of headache diagnosis and management was adequately covered in medical education [33]. Hence, the need to enhance educational content on migraines within medical training programs has been well recognized over the past two decades in the global context. Table 4 lists key perspectives from specialists on educational needs, gaps in migraine management, and critical findings from related studies.

**Table 4:** Summary of identified cross-sectional studies among specialists on educational needs and gaps in migraine management. AOAN: Asian Oceanian Association of Neurology; CGPR: calcitonin gene-related peptide; CPGs: clinical practice guidelines; EEG: electroencephalogram; GPs: general practitioners; HCPs: healthcare providers; ICHD: International Classification of Headache Disorders; MIDAS: Migraine Disability Assessment Scale; MOH: medication-overuse headache; NSAIDs: nonsteroidal anti-inflammatory drugs

Key insights collected	Key findings
Roxas A Jr et al., 2021 [30]: Delivery of care for migraine among member countries of Asian Oceanian Association of Neurology (AOAN)	
There is a critical need to allocate more time for lectures on primary headaches in medical schools, given the scarcity of clinical guidelines, subspecialty training, dedicated headache clinics, and patient advocacy organizations. Additionally, expanding the knowledge of GPs through short-term courses is essential.	Twenty-four specialists, each from all 21 member countries of AOAN and three other Asian countries, responded; 54% of countries have dedicated headache units. Ten out of 24 countries have CPGs for migraine management. All countries except Mongolia have at least one triptan available. Novel CGRP monoclonal antibodies for migraine prophylaxis are available mainly in high-income countries.
Roxas A Jr et al., 2022 [31]: Practice patterns of migraine management among neurologists in the Philippines	
The use of MIDAS scoring to assess migraine disability, as well as prescribing triptans and anti-nausea drugs, are not widespread practices among HCPs. There is a crucial need to advocate the use of ICHD criteria for migraine diagnosis to reduce unnecessary costs associated with EEG tests, which offer little additional diagnostic value. Furthermore, counselling on medication overuse is essential, as self-medication and overuse of NSAIDs are common due to the high cost of triptans for patients.	Two hundred and fifty-nine (56.7%) of 457 neurologists from the 2018 roster participated; 77% would request neuroimaging for patients with a history of >3 years of recurrent severe migraine. A headache diary was advised by 62.2%, and MIDAS was used by only 14.7%. Cautioning about MOH was done by 68.3%; 51% never or seldom prescribed a triptan for recurrent episodic moderate to severe migraine on the first outpatient consult. Only one out of three give anti-nausea medications for moderate to severe attacks of migraine. Only one out of three prescribe migraine prophylaxis for at least 12 weeks or longer.
Lu S-R et al., 2006 [32]: Practice pattern of migraine management among neurologists in Taiwan	
There is inadequate knowledge among HCPs regarding MOH, including unawareness that ergotamine, acetaminophen, and triptans are common causes. Additionally, adapting headache management guidelines to meet local educational needs is essential to improve patient care and treatment outcomes.	One hundred and twenty-three (23.2%) of 531 neurologists from the Taiwan Neurological Association participated. 88.5% felt that headache was an important part of their practice; 65.0% used neuroimaging to evaluate patients with severe headaches; 44.7% used EEG for headache evaluation; 12.2% prescribed preventive medications only when the patients had ≥14 headache days/month.
Kommineni M et al., 2005 [33]: Headache education perspectives from neurology residency program directors and chief residents in the United States	
Migraine was recognized as an important subject to teach in medical schools; however, there remains a significant impact of the pharmaceutical industry on migraine education and management.	One hundred and nineteen responded to the survey, including 75 chairs and 44 residency training directors from 95 institutions. Only 29% agreed or strongly agreed that headache diagnosis and management are adequately taught.

A 2020 survey by Aljunaid MA et al. in Jeddah revealed that PCPs show inadequate knowledge in diagnosing and managing CM. The survey, which had responses from 136 GPs, indicated inconsistent knowledge and generally inappropriate attitudes towards CM, with over half unable to identify duration criteria for CM. Additionally, nearly half of the PCPs acknowledged the issue of medication overuse in their patient population [34]. Similar findings from several studies in Europe [35, 36, 37] and the United States [38, 39] also point to a pressing global need for improved headache education and continuing medical education (CME) programs tailored explicitly for GPs to address these educational gaps (Table 5).

**Table 5:** Summary of identified cross-sectional studies on educational needs and gaps in migraine management among primary or front-line care providers AAN: American Academy of Neurology; CBT: cognitive behavioral therapy; CM: chronic migraine; CME: continuing medical education; GPs: general practitioners; HCPs: healthcare providers; MOH: medication-overuse headache; PCPs: primary care providers

Key insights collected	Key findings
Aljunaid MA et al., 2020 [34]: Level of knowledge of diagnosis and management of CM among PCPs in Jeddah	
PCPs have inadequate knowledge regarding the diagnosis and treatment of CM due to marked deficits in formal headache education for medical students. There is also a need to raise awareness about the risks of MOH. CME in migraine management is essential for PCPs to bridge educational gaps.	One hundred and thirty-six GPs responded to the survey. There was inconsistent knowledge and inappropriate attitudes towards CM (<70% of correct answers) for most items related to diagnosis and management. >50% could not identify the lower limits of CM duration, while only 45.6% recognized the issue of medication overuse in their patient communities.
Kristoffersen ES et al., 2021 [35]. Clinical knowledge of headache disorders among GPs across Norway	
GPs predominantly follow national guidelines while rarely referring to international ones. They, however, self-reported significant knowledge gaps in managing less common headaches such as cluster headaches, trigeminal autonomic cephalalgias, and MOH.	Three hundred and sixty-seven GPs responded from 130 CME groups. The GPs in this study had an average of 15 years of general experience. >50% of GPs reported headache management to be difficult. 1/3 adopted regular use of headache diaries; 52% were aware that different types of acute headache medication can lead to MOH, and 59% knew that prophylactic headache medication does not lead to MOH.
Ryvlin P et al., 2021 [36]: Chronic migraine (CM) patient management in primary care from 5 countries (European My-LIFE anamnesis survey)	
Patients with CM are not properly managed or referred to specialists in line with guideline recommendations. Imaging techniques are used by 50% of HCPs, indicating that patients are often subjected to unnecessary imaging procedures, which increases healthcare costs. Patient access to preventive drugs still remains a significant challenge.	Two hundred and one GPs from five countries (France, Germany, Italy, Spain, and the United Kingdom) were surveyed; 82% of GPs responsible for managing patients with disabling migraine or CM did not refer them to a specialist. Diagnosis in 61% of CM patients was made solely through anamnesis; 83% of participants from Germany stated that the main reason for referring their patients to a specialist is to give them access to preventive treatment.
Verhaak AMS et al., 2021 [38]: Knowledge and needs in migraine diagnosis and treatments regarding women’s healthcare providers in the United States	
The majority of HCPs do not routinely prescribe preventive medications for CM. This is also limited knowledge of headache-related red flags and a lack of thorough diagnostic workups to rule out secondary causes of headaches. Many are also less aware of medications that can result in MOH.	One hundred and fifteen women HCPs (including advanced practice registered nurses, physician assistants, and certified nurse midwives) responded to the survey. Only 6.3% were familiar with the AAN guideline on preventive treatment. Patient referrals were uncommon due to a lack of knowledge, provider availability, insurance issues, and patient scepticism. Providers indicated interest in education on migraine prevention and treatment (96.3%), migraine-associated disability (74.3%), and diagnostic testing (59.6%).
Gültekin M et al., 2018 [37]: Awareness of migraine among PCPs in Turkey	
Educational programs about migraines that are supported by complete educational materials are required for PCPs. Vocational training programs are needed to raise public awareness about migraine symptoms through official institutions.	Two hundred and sixty-six responses were received from GPs. Only 10.5% of physicians knew all the criteria for migraine diagnosis. 8.3% of the physicians had made a migraine diagnosis before; 80.8% of physicians reported that they had requested education about migraines. One-third perceived laboratory tests and brain screening as necessary for diagnosing migraines.
Minen MT et al., 2016 [39]: Knowledge and needs assessment of PCPs in the United States	
There was recognition of knowledge gaps, even among physicians from top academic centres. PCPs are less familiar with red flags or reasons that might warrant imaging. There is a crucial knowledge gap in preventive measures for migraines and awareness of guidelines and recommendations. There is a preference for educational mechanisms to increase contact with neurologists and headache specialists.	Eighty-three GPs completed the survey. Limited assessment by PCPs for comorbidities like depression (60%) and anxiety (50%) was demonstrated. There was a lack of imaging requests for the appropriate scenarios, and only a few GPs (27.8%) were aware of the AAN guidelines for prescribing preventive medications. One out of three were unaware that evidence-based non-pharmacologic therapy includes CBT, biofeedback, and relaxation training, and the remaining two out of three were aware but still not recommending it to their patients.

Identified deficits and proposed solutions for effective migraine management in Asia

In Asia, formal education on headache disorders at the medical school level is significantly lacking [22] [30]. This gap is compounded by the absence of comprehensive local clinical guidelines and a shortage of specialized headache training programs and clinics [30, 34]. Furthermore, the region faces a shortage of patient advocacy organizations, which is critical for supporting individuals with headaches [30]. This deficit in support exacerbates the widespread issue of MOH, precipitated by the OTC availability of pain medications [26].

Stress and sleep disturbances are frequently reported migraine triggers in Asia, with fatigue and weather changes common in Eastern Asia and fasting prevalent in Western Asia [40]. These regional differences highlight the importance of culturally sensitive educational programs that address specific triggers and lifestyle factors affecting migraine patients.

**Figure 1:**
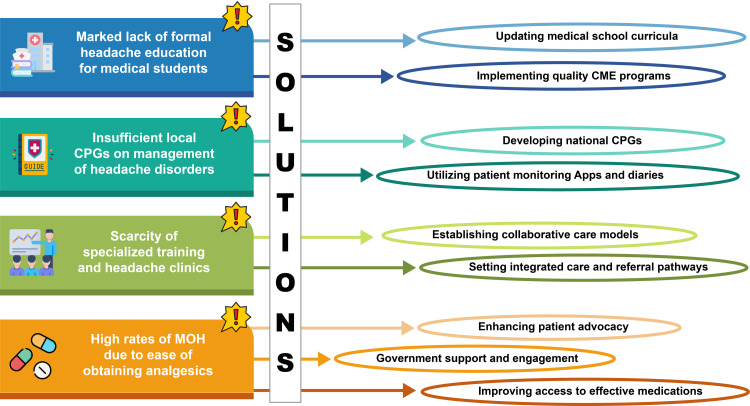
Challenges and solutions for effective migraine management in Asia There is a marked lack of formal headache education for medical students, along with insufficient local clinical guidelines and the scarcity of specialized training and headache clinics in Asia. There is also a paucity of patient advocacy groups in the region alongside the prevalence of MOH due to easy access to over-the-counter analgesics. Proposed immediate solutions emphasize the necessity of revising medical school curricula and specialized short-term courses to address these educational deficits for GPs, as well as the adoption of monitoring diaries or apps by patients. Apart from educational initiatives, developing national clinical practice guidelines, establishing collaborative care models and integrated care pathways, enhancing patient advocacy, and improving access to effective medications are essential to ensure quality management and sustainable support. CPGs: clinical practice guidelines; CME: continuing medical education; GPs: general practitioners; MOH: medication overuse headache. Image credits: This image has been created by the corresponding author, Sajita Setia.

There is a clear need for an improved educational framework to combat these challenges. This includes updating the medical school curriculum to incorporate dedicated headache management training, implementing effective CME programs, and developing short-term, focused courses for GPs to bridge the knowledge gap in formal training programs along with country- or region-specific clinical practice guidelines (CPGs). These steps are essential to equip clinicians with the necessary skills to manage and treat headache disorders effectively, ultimately improving patient care in the region. However, implementing the tailored educational framework faces potential barriers, including insufficient funding, fragmented healthcare systems, and inadequate coordination among HCPs [41,42]. Hence, a multifaceted approach is needed (Figure 1).

Key Solutions

- Updating medical school curricula: Incorporate dedicated headache management training of optimum duration to ensure new physicians are well-equipped to diagnose and treat migraines effectively [30,31].

- Implementing quality CME programs: Provide ongoing education for healthcare professionals, including short-term, focused courses for GPs to bridge knowledge gaps [30].

- Developing national CPGs: Establish standardized, evidence-based guidelines tailored to the local context to guide HCPs in diagnosing and managing migraines. The local CPGs should take into account the culturally specific triggers of migraine within the communities [30].

- Utilizing patient monitoring applications and headache diaries: Encourage the use of digital tools that allow patients to track their symptoms and treatment progress [43,44].

- Creating integrated care and referral pathways: Set up platforms where primary care physicians can consult with headache specialists for complex cases, enhancing access to expert advice and reducing delays in patient care [41,42].

- Establishing collaborative care models: Form multidisciplinary teams, including neurologists, primary care physicians, and mental health professionals, to provide comprehensive care for migraine patients [41].

- Enhancing patient advocacy: Support the development and availability of patient advocacy organizations to provide education, resources, and support to individuals with migraines, including education on the risks of self-medication and MOH [41].

- Government support and engagement: Encourage the collection of local epidemiological data and studies on the cost and burden of migraine management to inform policy-making and resource allocation [41].

- Improving access to effective medications: Address inadequate reimbursement and cost barriers that lead clinicians to choose migraine medications based on availability and price rather than efficacy and safety. For example, despite triptans being more effective and safer, ergotamine continues to be commonly used in low- and middle-income countries (LMICs) due to cost considerations. Enhancing reimbursement policies and making effective medications more affordable can improve treatment outcomes [41].

Steiner TJ et al. have proposed a three-tiered system for headache healthcare provision, which could be very useful, especially in LMICs [18, 45]. It has been advocated by the global campaign 'Lifting the Burden against Headache’ in collaboration with the European Headache Federation. This system is reliant on improved training of HCPs at level one and reserves limited specialist care for individuals with the most complex and difficult-to-treat headache disorders.

- Level 1 providers: Clinicians such as GPs and other PCPs handle the majority of headache cases, including common primary headaches and the initial identification of secondary headaches. After undergoing a focused training program, these providers are equipped to manage about 90% of headache sufferers, ensuring that only the more complex cases are referred upwards.

- Level 2 providers: Physicians with additional training in headache medicine (although not necessarily neurologists) handle more complicated primary headaches and some secondary headaches that are beyond the scope of Level 1 providers but not so severe as to require specialist intervention. This level should aim to manage 9% out of the remaining 10% of cases.

- Level 3 providers: Specialist neurologists treat the most complex or rare primary and secondary headaches requiring specialized care (the remaining 1% of cases only).

Collaboration among organizations is essential to promote quality neurology care and better brain health in the region. The Asian Regional Consortium for Headache (ARCH) exemplifies such collaborative efforts through partnerships between LMICs and high-income nations in this region [46]. As of 2022, ARCH has expanded its membership to a total of 19 member countries: China, India, Japan, Thailand, Taiwan, Malaysia, Laos, Myanmar, Nepal, the Philippines, South Korea, Singapore, Australia, New Zealand, Bangladesh, Pakistan, Indonesia, and Sri Lanka. The ARCH aims to work synergistically with the IHS, the World Federation of Neurology, and the World Health Organization to promote quality neurology and enhance brain health for patients with headache disorders across Asia and Oceania [46]. However, while collaboration forms the backbone of effective healthcare delivery, implementing innovative educational methods in CME is equally critical for the practical application of evidence-based medicine (EBM) in patient care. For this, CME programs need to transcend traditional teaching methods through implementation science by integrating the principles of cognitive psychology and information mastery [47,48]. Implementation science in this context refers to the application of methods that promote the integration of EBM and policies into real-world clinical practice improvements for migraine diagnosis and management in the region [49].

Limitations

We adopted a semi-systematic research approach instead of a more rigorous systematic review methodology. One limitation of this approach is that it may not provide a comprehensive and systematic analysis of the available literature, unlike a systematic review. However, our choice of a semi-systematic, narrative-style review was driven by the need for a more flexible and interpretative analysis of a wide array of heterogeneous studies to match our extremely broad research question on unmet needs in education and clinical practice [21]. This approach allowed us to draw broader insights and understand gaps and challenges in migraine management education in the Asian healthcare setting within a global context. Despite its limitations in capturing all-inclusive data, the narrative review methodology enabled us to explore and synthesize key themes and patterns effectively, which is crucial for a topic with varied global and regional dimensions.

## Conclusions

Our review identifies a critical deficiency in the formal education and training of healthcare professionals for headache disorders. The need for comprehensive training in headache medicine within medical curricula and residency programs is a global concern with specific implications for Asia's diverse healthcare landscape. This shortfall is a major barrier to effective headache care, often leading to suboptimal treatment and management. Addressing this requires systematic efforts to identify and bridge knowledge gaps in headache care among healthcare providers. Enhancing education at all levels, starting with undergraduate medical curricula and continuing ongoing education for GPs and specialists, is crucial. Additionally, public education on self-medication practices is crucial to mitigate issues like medication overuse and MOH. Improving educational standards in migraine management is vital for elevating patient care and addressing this significant public health challenge. However, aside from the educational initiatives, integrating and coordinating systems of care (where primary and specialist services complement and support each other) are essential for improvement in patient care. The use of robust and quality education, along with comprehensive referral and integrated care protocols, will enable continuity of care between different levels of the healthcare system. Collaboration, communication, and cooperation between HCPs as well as organizations are essential to improve the quality of life and productivity for patients with migraine in the region.

## References

[1] (2020). Global burden of 369 diseases and injuries in 204 countries and territories, 1990-2019: a systematic analysis for the Global Burden of Disease Study 2019. Lancet.

[2] Steiner TJ, Stovner LJ, Jensen R, Uluduz D, Katsarava Z (2020). Migraine remains second among the world's causes of disability, and first among young women: findings from GBD2019. J Headache Pain.

[3] (2024). Global incidence, prevalence, years lived with disability (YLDs), disability-adjusted life-years (DALYs), and healthy life expectancy (HALE) for 371 diseases and injuries in 204 countries and territories and 811 subnational locations, 1990-2021: a systematic analysis for the Global Burden of Disease Study 2021. Lancet.

[4] Saylor D, Steiner TJ (2018). The global burden of headache. Semin Neurol.

[5] Chen ZF, Kong XM, Yang CH, Li XY, Guo H, Wang ZW (2024). Global, regional, and national burden and trends of migraine among youths and young adults aged 15-39 years from 1990 to 2021: findings from the global burden of disease study 2021. J Headache Pain.

[6] Ge R, Chang J (2023). Disease burden of migraine and tension-type headache in non-high-income East and Southeast Asia from 1990 to 2019. J Headache Pain.

[7] Nakatani H (2023). Ageing and shrinking population: the looming demographic challenges of super-aged and super-low fertility society starting from Asia. Glob Health Med.

[8] Steiner TJ, Stovner LJ (2023). Global epidemiology of migraine and its implications for public health and health policy. Nat Rev Neurol.

[9] Wang SJ (2003). Epidemiology of migraine and other types of headache in Asia. Curr Neurol Neurosci Rep.

[10] Takeshima T, Wan Q, Zhang Y (2019). Prevalence, burden, and clinical management of migraine in China, Japan, and South Korea: a comprehensive review of the literature. J Headache Pain.

[11] Wijeratne T, Tanprawate S, Singh L, Chen SP, Martelletti P (2024). Unveiling a groundbreaking alliance: the inaugural collaboration between the Asian Regional Consortium of Headache and The Journal of Headache and Pain. J Headache Pain.

[12] Fuh JL, Wang SJ, Lu SR (2008). Impact of migraine on the employed labor force in Taiwan. J Chin Med Assoc.

[13] Yu S, Liu R, Zhao G (2012). The prevalence and burden of primary headaches in China: a population-based door-to-door survey. Headache.

[14] Ong JJY, Patnaik D, Chan YC, Simon O, Finkelstein EA (2020). Economic burden of migraine in Singapore. Cephalalgia Rep.

[15] Tang CH, Chen YC, Ng K, Wang SJ (2013). A retrospective matched case-control study on medical costs of refractory migraine in Taiwan. Headache.

[16] Leonardi M, Martelletti P, Burstein R (2024). The World Health Organization Intersectoral Global Action Plan on Epilepsy and Other Neurological Disorders and the headache revolution: from headache burden to a global action plan for headache disorders. J Headache Pain.

[17] Martelletti P, Leonardi M, Ashina M (2023). Rethinking headache as a global public health case model for reaching the SDG 3 HEALTH by 2030. J Headache Pain.

[18] Mortel D, Kawatu N, Steiner TJ, Saylor D (2022). Barriers to headache care in low- and middle-income countries. eNeurologicalSci.

[19] Luciani M, Negro A, Spuntarelli V, Bentivegna E, Martelletti P (2021). Evaluating and managing severe headache in the emergency department. Expert Rev Neurother.

[20] Blumenfeld A, Dueland AN, Evers S, Jenkins B, Martelletti P, Sommer K (2022). Practical insights on the identification and management of patients with chronic migraine. Pain Ther.

[21] Snyder H (2019). Literature review as a research methodology: an overview and guidelines. J Bus Res.

[22] Ong JJ, Chan YC (2017). Medical undergraduate survey on headache education in Singapore: knowledge, perceptions, and assessment of unmet needs. Headache.

[23] Gallagher RM, Alam R, Shah S, Mueller L, Rogers JJ (2005). Headache in medical education: medical schools, neurology and family practice residencies. Headache.

[24] Young WB, Rosen NL, Sheftell F (2010). Headache education for the medical student. Headache Care, Research and Education Worldwide: Frontiers in Headache Research Series.

[25] Steiner TJ, Stovner LJ, Dua T (2011). Time to act on headache disorders. J Headache Pain.

[26] Tawakul AA, Aldharman SS, Al-Rabiah NM (2023). Assessment of barriers and challenges in headache education among neurology residents in Saudi Arabia. Cureus.

[27] Beltramone M, Redon S, Fernandes S, Ducros A, Avouac A, Donnet A (2022). The teaching of headache medicine in France: a questionnaire-based study. Headache.

[28] Do TP, Dømgaard M, Stefansen S, Kristoffersen ES, Ashina M, Hansen JM (2022). Barriers and gaps in headache education: a national cross-sectional survey of neurology residents in Denmark. BMC Med Educ.

[29] Ahmed ZA, Faulkner LR (2016). Headache education in adult neurology residency: a survey of program directors and chief residents. Headache.

[30] Roxas A Jr, Quiles LE, Wang SJ (2021). Delivery of care for migraine in the Asian Oceanian region: a cross-sectional study. Cephalalgia.

[31] Roxas Jr AA, Quiles LE, Diamante P (2022). The practice patterns of migraine management among neurologists in the Philippines - a cross-sectional survey. Neurol Asia.

[32] Lu SR, Wang SJ, Fuh JL (2006). The practice pattern of migraine management among neurologists in Taiwan. Cephalalgia.

[33] Kommineni M, Finkel AG (2005). Teaching headache in America: survey of neurology chairs and residency directors. Headache.

[34] Aljunaid MA, Jamal HH, Mubarak AA, Bardisi W (2020). Levels and determinants of knowledge about chronic migraine diagnosis and management among primary health-care physicians in ministry of health, Jeddah 2019. J Family Med Prim Care.

[35] Kristoffersen ES, Faiz KW, Hansen JM, Tronvik EA, Frich JC, Lundqvist C, Winsvold BS (2021). The management and clinical knowledge of headache disorders among general practitioners in Norway: a questionnaire survey. J Headache Pain.

[36] Ryvlin P, Skorobogatykh K, Negro A (2021). Current clinical practice in disabling and chronic migraine in the primary care setting: results from the European My-LIFE anamnesis survey. BMC Neurol.

[37] Gültekin M, Balci E, İsmaİLOĞUL S (2018). Awareness of migraine among primary care physicians in Turkey: a regional study. Noro Psikiyatr Ars.

[38] Verhaak AM, Williamson A, Johnson A (2021). Migraine diagnosis and treatment: a knowledge and needs assessment of women's healthcare providers. Headache.

[39] Minen MT, Loder E, Tishler L, Silbersweig D (2016). Migraine diagnosis and treatment: a knowledge and needs assessment among primary care providers. Cephalalgia.

[40] Iba C, Ohtani S, Lee MJ (2023). Migraine triggers in Asian countries: a narrative review. Front Neurol.

[41] Ashina M, Katsarava Z, Do TP (2021). Migraine: epidemiology and systems of care. Lancet.

[42] Tham TY, Tran TL, Prueksaritanond S, Isidro JS, Setia S, Welluppillai V (2018). Integrated health care systems in Asia: an urgent necessity. Clin Interv Aging.

[43] Chen X, Luo Y (2023). Digital therapeutics in migraine management: a novel treatment option in the COVID-19 era. J Pain Res.

[44] Jonker L, Fitzgerald L, Vanderpol J, Fisher S (2022). Digital diary app use for migraine in primary care: prospective cohort study. Clin Neurol Neurosurg.

[45] Steiner TJ, Jensen R, Katsarava Z (2021). Structured headache services as the solution to the ill-health burden of headache: 1. rationale and description. J Headache Pain.

[46] Wijeratne T, Ravishankar K (2023). ARCH is bringing Asia closer to the rest of the world. Brain Sci.

[47] Setia S, Loo E, Shinde SP, Singh M, Wong CH, Thakkar K (2024). Redefining the role of medical affairs professionals as innovators and leaders in industry-led medical education. Pharmaceut Med.

[48] Schmidt HG, Mamede S (2020). How cognitive psychology changed the face of medical education research. Adv Health Sci Educ Theory Pract.

[49] Chu F (2024). Implementation science: why should we care?. J Med Libr Assoc.

